# Intradermal delivery of a synthetic DNA vaccine protects macaques from Middle East respiratory syndrome coronavirus

**DOI:** 10.1172/jci.insight.146082

**Published:** 2021-05-24

**Authors:** Ami Patel, Emma L. Reuschel, Ziyang Xu, Faraz I. Zaidi, Kevin Y. Kim, Dana P. Scott, Janess Mendoza, Stephanie Ramos, Regina Stoltz, Friederike Feldmann, Atsushi Okumura, Kimberly Meade-White, Elaine Haddock, Tina Thomas, Rebecca Rosenke, Jamie Lovaglio, Patrick W. Hanley, Greg Saturday, Kar Muthumani, Heinz Feldmann, Laurent M. Humeau, Kate E. Broderick, David B. Weiner

**Affiliations:** 1 Vaccine & Immunotherapy Center, The Wistar Institute, Philadelphia, Pennsylvania, USA.; 2 Department of Pharmacology, Perelman School of Medicine, University of Pennsylvania, Philadelphia, Pennsylvania, USA.; 3 Rocky Mountain Veterinary Branch, Division of Intramural Research, National Institutes of Allergy and Infectious Diseases, NIH, Hamilton, Montana, USA.; 4 Inovio Pharmaceuticals Inc., Plymouth Meeting, Pennsylvania, USA.; 5 Laboratory of Virology, Division of Intramural Research, National Institutes of Allergy and Infectious Diseases, NIH, Hamilton, Montana, USA.

**Keywords:** Infectious disease, Vaccines, Adaptive immunity

## Abstract

Emerging coronaviruses from zoonotic reservoirs, including severe acute respiratory syndrome coronavirus (SARS-CoV), Middle East respiratory syndrome coronavirus (MERS-CoV), and severe acute respiratory syndrome coronavirus 2 (SARS-CoV-2), have been associated with human-to-human transmission and significant morbidity and mortality. Here, we study both intradermal and intramuscular 2-dose delivery regimens of an advanced synthetic DNA vaccine candidate encoding a full-length MERS-CoV spike (S) protein, which induced potent binding and neutralizing antibodies as well as cellular immune responses in rhesus macaques. In a MERS-CoV challenge, all immunized rhesus macaques exhibited reduced clinical symptoms, lowered viral lung load, and decreased severity of pathological signs of disease compared with controls. Intradermal vaccination was dose sparing and more effective in this model at protecting animals from disease. The data support the further study of this vaccine for preventing MERS-CoV infection and transmission, including investigation of such vaccines and simplified delivery routes against emerging coronaviruses.

## Introduction

Middle East respiratory syndrome (MERS) coronavirus (MERS-CoV) is a positive-sense, single-stranded RNA coronavirus that infects the lower and upper respiratory tract, causing a viral pneumonia characterized by acute respiratory symptoms, such as fever, aches, shortness of breath, sore throat, cough, diarrhea, and vomiting (1). Since 2012, there have been 2566 laboratory-confirmed cases and 882 MERS-CoV–associated deaths (34.4% case fatality rate) (2). Human cases are frequently associated with close contact of infected camels; however, human-to-human transmission, nosocomial infections, and travel-associated cases have been observed. MERS-CoV has therefore become a global health priority concern. The 2015 South Korean outbreak originated from a single traveler who returned home from the Middle East. In total, 186 people were infected during the South Korean outbreak, with 36 MERS-associated fatalities (3) and a significant impact on the healthcare system. This outbreak highlights the importance of rapid infection control for emerging coronaviruses and other infectious diseases. The urgent need for accelerated vaccine development has become critical in light of the ongoing severe acute respiratory syndrome coronavirus 2 (SARS-CoV-2) pandemic, a betacoronavirus related to MERS-CoV.

DNA vaccines are a nonlive, noninfectious platform that are re-administrable, easily scalable for manufacturing, have an established safety and tolerability profile, and are heat stable (4, 5). We previously described the rapid development of an anti-MERS synthetic DNA vaccine encoding a full-length MERS-CoV spike (S) antigen, which induced robust humoral and cellular immune responses and protected rhesus macaques from MERS-CoV challenge (6). This MERS DNA vaccine candidate (INO-4700/GLS-5300), delivered by intramuscular (i.m.) administration, was found to be safe and tolerable with a 3-dose injection regimen in a recently completed human phase I study (7) and is currently in expanded studies of a phase I/IIa trial in South Korea.

Further study of low-dose delivery with shortened dosing regimens is important to rapidly induce protective immunity, particularly during an emerging outbreak (8). Here, we describe i.m. and intradermal (i.d.) delivery, immunogenicity, and protective efficacy of the MERS DNA vaccine candidate INO-4700, using an abbreviated 2-dose immunization regimen in rhesus macaques. We observed induction of strong antibody titers against the full-length S protein as well as the receptor-binding domain (RBD), S1, and S2 regions of the S protein. We also observed induction of neutralizing antibody responses and cellular immune responses. Finally, the animals were challenged and the effect of the vaccination on infection against vigorous MERS-CoV challenge in nonhuman primates (NHPs) was studied. Macaques receiving this 2-dose vaccine demonstrated lower viral loads with protection of the lung from inflammation, protection against elevated cytokine levels, and, most importantly, protection against clinical disease symptoms such as breathing difficulties. Even low-dose i.d. delivery afforded comparable efficacy to higher dose i.d. and i.m. regimens, and both i.d. immunizations exhibited improved disease control compared with i.m. vaccination. The data support further evaluation of simple dose-sparing i.d.-delivered DNA vaccination regimens against MERS-CoV. These advances have important applicability for similar DNA vaccines and i.d. delivery against other emerging betacoronaviruses, such as SARS-CoV-2, as well as for future emerging infectious diseases.

## Results

### Immunogenicity of i.d. delivered MERS DNA vaccine.

**Figure 1:**
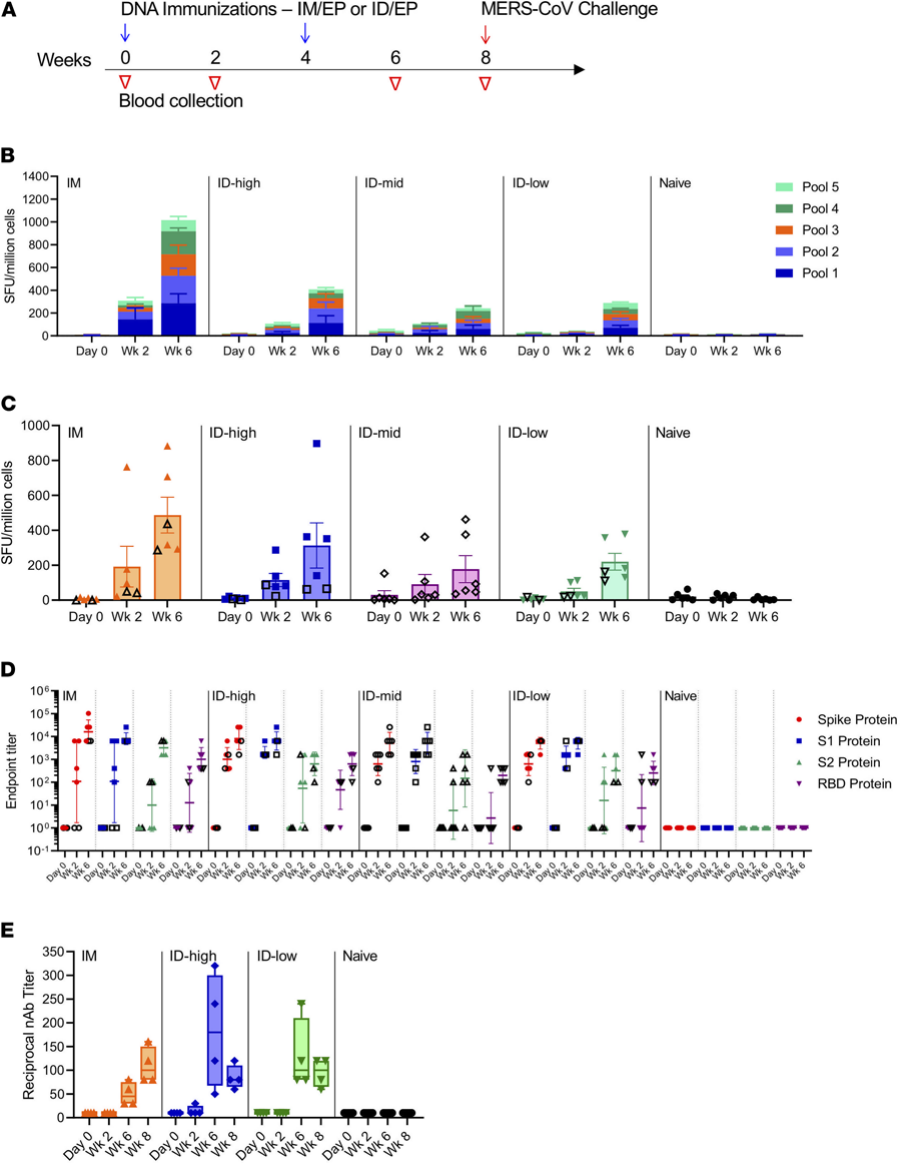
Study timeline and immune responses induced by MERS DNA vaccine. (**A**) Immunization and blood collection timeline. Rhesus macaques (*n* = 6) were immunized i.m. with 1 mg or i.d. with 2 mg (i.d.-high), 1 mg (i.d.-mid), or 0.2 mg (i.d.-low) of MERS DNA vaccine at the indicated time points. Control animals were not vaccinated. Blood was collected at the indicated time points for immune analysis. (**B**) Vaccine-induced antigen-specific IFN-γ ELISPOT responses represented by peptide pool. PBMCs from each animal at each time point were stimulated with peptide pools covering the MERS spike protein, and numbers of cells secreting IFN-γ were counted. Group average spot-forming units (SFU) per million cells are presented for each peptide pool. Error bars represent SEM. (**C**) Protein stimulated antigen-specific IFN-γ ELISPOT responses. PBMCs from each animal at each time point were stimulated with recombinant full-length MERS S protein, and numbers of cells secreting IFN-γ were counted. Individual values are shown by the symbols with the group average indicated by the bar. Error bars represent mean ± SEM. Animals represented with closed symbols were challenged with MERS-CoV 4 weeks after final immunization. Open symbols depict the responses for animals that were not selected for challenge. (**D**) Vaccine-induced MERS spike–specific endpoint binding titers. Sera from each animal at each time point were evaluated for their ability to bind to full-length MERS S, S1, S2, and RBD proteins. Endpoint titers for individual animals are shown with the geometric mean and 95% confidence interval indicated by the bars. Error bars represent mean ± SEM. Animals represented with closed symbols were challenged with MERS-CoV 4 weeks after final immunization Open symbols depict responses for animals that were not selected for challenge. (**E**) Vaccine-induced neutralizing antibody titers in challenged animals (*n* = 4/vaccinated groups, *n* = 6/naive). Sera were evaluated for their ability to neutralize MERS-CoV. Reciprocal neutralizing antibody (nAb) titers are shown, with boxed indicating 25th percentile, median, and 75th percentile, and whiskers showing the minimum and maximum values.

Very recent advances in formulations for i.d. delivery of synthetic DNA vaccines with adaptive electroporation (EP) have significantly improved the generation of antigen-specific immune responses, including long-term antibody and T cell responses induced in human trials, with responses persisting at least 1 year after vaccination (9–11). Delivery of DNA vaccines i.d. is tolerable, simple to administer, and is potentially more immunogenic than i.m. delivery when given at the same dose in recent clinical studies (7, 9, 10). Therefore, we evaluated the efficacy of our previously described synthetic MERS DNA vaccine (6), which had been studied in NHP using an i.m. 3-dose immunization regimen. Here, we studied an abbreviated 2-dose i.d. immunization regimen and compared this approach with i.m. delivery. Rhesus macaques (*n* = 6/group) were first administered either a 0.2 mg dose (i.d.-low), a 1 mg dose (i.d.-mid), or a 2 mg dose (i.d.-high) of the MERS DNA vaccine by i.d. injection followed by adaptive EP. The i.m. group (*n* = 6) received a 1.0 mg dose. All vaccinated groups received a 2-dose regimen, spaced at a 4-week interval (Figure 1A). The control group (*n* = 6) was not vaccinated.

Cellular and humoral immune responses were assayed following each immunization. Following the immunization studies, we selected 3 of the groups and 4 of the animals from each of the selected groups for MERS viral challenge, based on space limitations. We analyzed both humoral and cellular responses, as the role of both adaptive immune compartments may be important for viral clearance and recovery from infection, as has been described for both SARS-CoV and MERS-CoV (12, 13) and suggested by recent studies of human immune responses in convalescent patients with SARS-CoV-2 (14, 15). We analyzed the induction of T cell responses by IFN-γ ELISpot 2 weeks after each immunization. T cell responses against peptide pools spanning the full-length S protein were readily detected in 6 of 6 NHPs in the i.m. group (432–2067 spot-forming units [SFU]/million PBMCs), 6 of 6 NHPs in the i.d.-high-dose group (73–1018 SFU/million PBMCs), 6 of 6 NHPs in the i.d.-mid dose group (52–857 SFU/million PBMCs), 6 of 6 NHPs in the i.d.-low-dose group (160–422 SFU/million PBMCs), and 0 of 6 NHPs in the naive control group (2–33 SFU/million PBMCs) after 2 DNA immunizations (Figure 1B and Supplemental Figure 1; supplemental material available online with this article; https://doi.org/10.1172/jci.insight.146082DS1). Additionally, IFN-γ ELISpot assays were performed using full-length recombinant S protein for stimulation as a tool to address rapid vaccine evaluation during an outbreak in the absence of synthetic peptide pools. Although fewer total spots were observed, on average, strong T cell responses were induced in all groups following a similar trend to those observed with peptide pools (Figure 1C), supporting the full-length antigen study as an additional assay tool in evaluation of vaccine immunogenicity.

To address the question of antibody responses following in vivo processing of a full-length spike protein antigen, we assayed antibody endpoint titers against the full-length S as well as S1, S2, and RBD by total IgG binding ELISA. After 1 immunization, 67% (4 of 6) of i.m. animals, 100% (6 of 6) of i.d.-high animals, 100% (6 of 6) of i.d.-mid animals, and 100% (6 of 6) of i.d.-low animals seroconverted to full-length S and S1 proteins. After 1 immunization, 50% (3 of 6) of i.m. animals, 67% (4 of 6) of i.d.-high animals, 33% (2 of 6) of i.d.-mid animals, and 50% (3 of 6) of i.d.-low animals seroconverted to S2 protein. After 1 immunization, 50% (3 of 6) of i.m. animals, 83% (5 of 6) of i.d.-high animals, 17% (1 of 6) of i.d.-mid animals, and 33% (2 of 6) of i.d.-low animals seroconverted to RBD protein. After 2 immunizations, all vaccinated animals seroconverted to full-length S, S1, S2, and RBD proteins, except for 1 animal in the i.d.-mid group that did not seroconvert to S2 protein (Figure 1D). Two weeks after the second immunization, geometric mean endpoint titers in all groups were approximately 10^4^ for both full-length S and S1 proteins. Geometric mean endpoint titers in all groups were approximately 10^2^ to 10^3^ for S2 and RBD, with a trend for slightly higher titers in the i.m. group, though there were no significant differences in endpoint titer values between vaccine groups. Overall, the antibody responses induced in this study demonstrate the consistency of synthetic DNA vaccination and robust induction of antibody responses by the simple i.d. delivery. Notably, responses were also robust in the low-dose (0.2 mg) i.d.-delivered MERS DNA vaccine group (Figure 1D).

Eighteen animals were selected for challenge with MERS-CoV of the 30 total animals, due to funding and space limitations. Based on the ELISpot and endpoint binding antibody titer data available at the time, a total of 12 vaccinated animals and the 6 naive control animals were moved into a challenge study (animals that were not selected for challenge are indicated by open shapes in Figure 1, C and D). There is no statistical difference regarding immune responses between the animals in each group that were challenged compared with those that were not challenged. Because the i.d.-low group exhibited robust immunogenicity, we wanted to compare its challenge outcome to the i.d.-high group, so 4 animals each from the i.d.-high and i.d.-low groups were chosen for the challenge. Four animals from the i.m. group served as a comparison with previous studies, which were 3-dose immunization studies (6).

Neutralizing antibody titers for the challenged animals were assayed using MERS-CoV EMC/2012 (Figure 1E). Neutralizing activity was detected in the sera after the boost, peaking at week 6, with average titers of 50, 170, and 130 for i.m., i.d.-high, and i.d.-low groups, respectively. By week 8, all vaccinated groups had comparable neutralizing antibody titers, demonstrating that similar binding and neutralizing antibody titers could be induced by low-dose (0.2 mg) i.d. vaccination as compared with higher doses (1.0 mg i.m. vaccination and 2.0 mg i.d. vaccination). Delivery i.d. appears dose sparing based on this comparison, and a similar observation has recently been reported for an HIV DNA vaccine studied in the clinic, which was delivered by the Cellectra i.d. EP approach (11).

### Challenge outcome of i.d. versus i.m. MERS DNA vaccine regimens.

**Figure 2:**
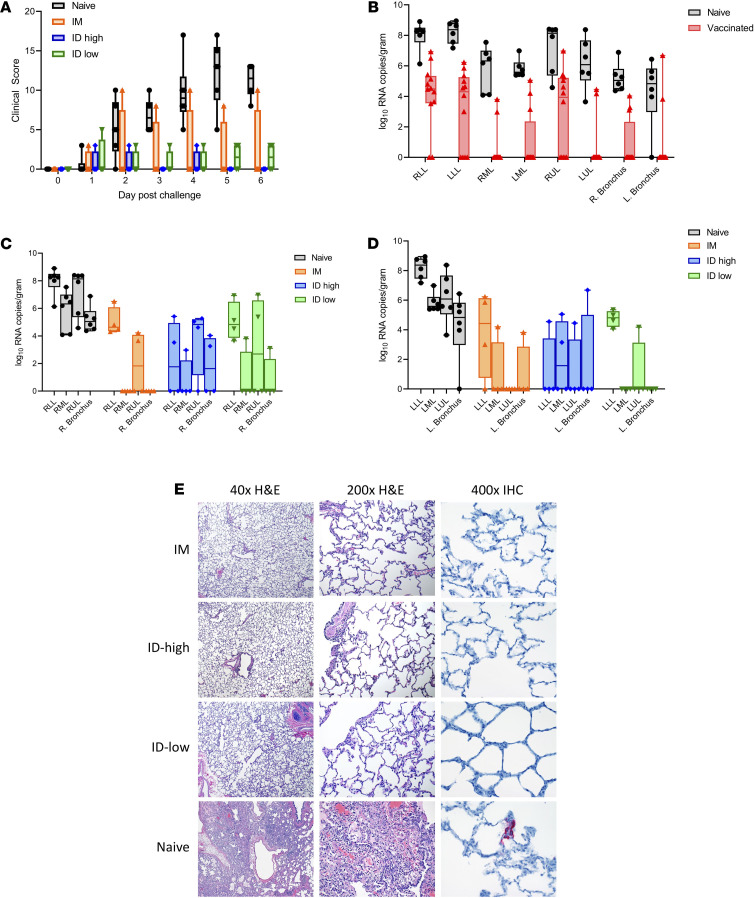
Postchallenge pathology prevented by MERS DNA vaccine. (**A**) Clinical scores for each group after challenge. Animals were scored for visible signs of disease daily following challenge, with increasing scores indicating more severe symptoms. (**B**) Viral loads in vaccinated versus naive animals. (**C** and **D**) Viral loads in various tissues for each group after challenge. The viral load at day 6 after challenge in respiratory tissues and lymph nodes was measured by RT-PCR. Individual animals are included in the box-and-whisker plots, with whiskers showing the minimum and maximum values. (**E**) Representative H&E-stained and IHC-stained lung tissue sections from animals in each vaccination group day 6 after challenge. Vaccinated animals demonstrate essentially normal lung parenchyma. The naive animal shows moderate interstitial pneumonia. Viral antigen was detected by IHC (pink stain) in 4 of 6 control animals, but none of the immunized animals. Original magnification, ×40 (H&E, left); ×200 (H&E, right); ×400 (IHC).

Macaques were challenged by inoculation with 7 × 10^6^ median tissue culture infectious dose (TCID_50_) of MERS-CoV EMC/2012 strain through rigorous installation via a *combination* of intratracheal, intranasal, oral, and ocular administrations, as previously established (16, 17). NHPs were monitored for clinical signs of disease and also received chest x-rays on days 0, 1, 3, 5, and 6 after challenge, before they were euthanized and necropsied on day 6 for lung pathology and viral load determination. All immunized animals, except 1 i.m. animal (11 of 12), had a major reduction in clinical signs of disease as compared with the control group (Figure 2A and Supplemental Table 1) showing significant disease protection. A upE qRT-PCR assay was performed to detect viral loads present in the collected lung tissue. Overall, compared with the unvaccinated animals, all MERS DNA vaccine groups exhibited log reductions in viral loads across all regions of the lower airways (Figure 2B). Significantly decreased viral loads were observed in all vaccinated groups compared with control animals in the right bronchus, right middle lung, right lower lung, left upper lung, left middle lung, and left lower lung lobes (*P* values are listed in Supplemental Table 2). Four of four i.d.-low and three of four i.m. and i.d.-high animals had no viral loads in the left bronchus, and animals trended toward decreased loads in the right upper lung (Figure 2, C and D; *P* values listed in Supplemental Table 3). Minimal virus was detected in the routes of installation challenge. It is likely that residual virus from the installation was being detected in these tissues, as 2 animals were still positive in the conjunctiva (ocular administration route), a nonrespiratory tissue, at day 6. In both the vaccinated and control groups, radiographic signs of disease were minimal. Lung tissues from all challenged animals were examined with H&E staining and IHC against MERS-CoV antigen to evaluate virus-induced pathology (Figure 2E). Histological evidence of mild focal interstitial pneumonia was observed in 5 of 12 animals in the vaccinated group, with multifocal moderate interstitial pneumonia in all 6 naive macaques. All 6 animals in the control group eventually developed multiple symptoms of disease, including difficulty breathing, as did 1 animal in the i.m. vaccinated group. No animals in the i.d. groups exhibited symptoms associated with lung impact in the challenge course of study. All of the control animals showed lung disease symptoms during the challenge course as well as other symptoms. Finally, MERS-CoV antigen was detected through IHC in 4 of 6 lung specimens from unvaccinated macaques but was not observed in any vaccinated macaques (Figure 2E).

**Figure 3:**
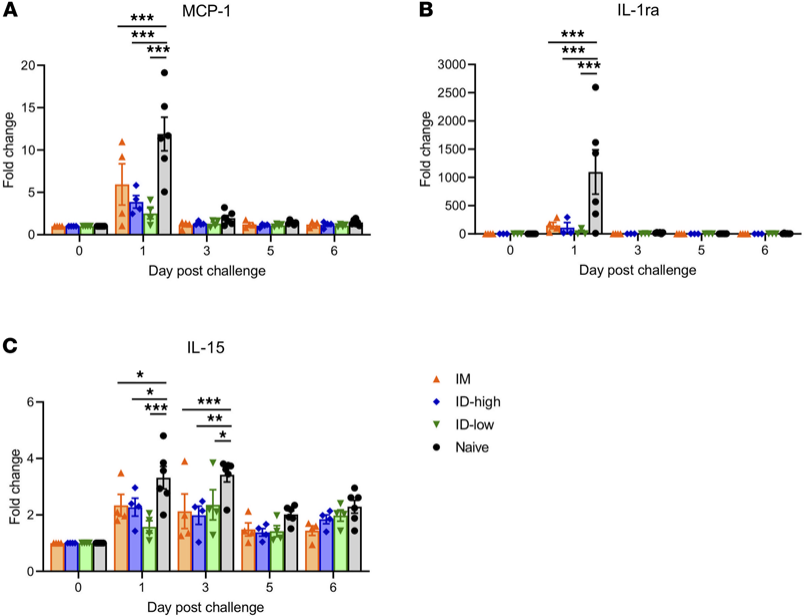
Serum cytokine changes after challenge. (**A**) MCP-1, (**B**) IL-1ra, and (**C**) IL-15 cytokine levels in serum after challenge. Individual values are shown by the symbols, with the group average indicated by the bar; error bars represent mean ± SEM. *P < 0.05, **P < 0.01, ***P < 0.001 compared with the naive control. Nonparametric Mann-Whitney test, adjusted for multiple comparisons using a Bonferroni correction, was used for statistical analysis.

After challenge, sera were screened against a Luminex 23-cytokine panel (G-CSF, GM-CSF, IFN-γ, IL1-β, IL-1ra, IL-2, IL-4, IL-5, IL-6, IL-8, IL-10, IL-12/23p40, IL-13, IL-15, IL-17A, IL-18, MCP-1, MIP-1α, sCD40L, TGF-α, TNF-α, and VEGF) to assess potential inflammation impact. In control animals, we observed a significant increase of early innate cytokines MCP-1, IL-1ra, and IL-15. By comparison, this increase was abrogated in all vaccinated animals compared with unvaccinated controls (Figure 3). No significant changes in other cytokines were observed or the cytokine levels were below the limit of detection of the assay, supporting a lack of inflammation enhancement by this panel of immune markers.

## Discussion

In the last twenty years, 3 new CoV have emerged from zoonotic reservoirs (MERS, SARS, and SARS-CoV-2). There are no licensed vaccines to prevent coronavirus infections in people; however, important products are advancing in this space. Vaccine candidates that are simple to deliver, well tolerated, do not induce anti-vector immunity, and that can be readily administered in resource limited settings could be important. There have been a few other vaccine candidates evaluated in NHP challenge studies for MERS. These include an rRBD-plus-adjuvant-vaccine approach using 3 immunizations reported by Lan et al., which induced partial protection in a short-term, 3-day challenge NHP model (18). A study by L. Wang et al., using combinations of DNA vaccines and protein boosts, showed limited vaccine effect on infection by CT scan read out (19). A recent study by van Doremalen et al. tested a MERS-CoV spike recombinant Chimpanzee Ad (ChAdOx) vaccine (20) in a similar challenge model to the one presented here and similar to our earlier i.m. DNA immunization challenge (6). The ChAdOx vaccine was tested in 1- or 2-dose regimens. Both dose regimens could effect viral disease and viral load, particularly in the lower respiratory tract, with the single-dose regimen exhibiting a smaller protective effect with limited effect on pathogenesis compared with the 2-dose regimen. Data from these reports are illustrative of the utility of this particular multiple route–challenge NHP model developed at Rocky Mountain Labs (RML) for vaccine testing. It is reproducible and provides broad tissue sampling as well as disease read outs (6, 16, 17, 20).

Here, we investigated the immunogenicity and protective efficacy of an i.d.-delivered synthetic MERS DNA vaccine using a shortened 2-dose immunization schedule and compared this to an i.m. delivered 2-dose DNA vaccine formulation. Immune analysis compared 3 different vaccine doses for their immune potency by i.d. delivery in parallel with i.m. delivery. The MERS DNA vaccines induced antibody responses against all regions of the S protein and robust neutralizing antibodies. Cellular immune responses were induced in all animals, which may be important for clearance of virally infected cells, limiting pathogenesis, and reducing viral loads. Vaccines that drive both antibody and T cell immunity could be important for preventing asymptomatic spread and protecting the lower airway, thus mitigating disease. For challenge, we downselected animals to focus on vaccine groups from the i.d.-low, i.d.-high, and i.m. immunization groups, and due to cost constraints, we were limited in the number of animals in each challenge group, although the immune spread was overlapping. Challenge outcome showed that all 3 vaccination groups protected rhesus macaques against MERS-CoV EMC/2012 challenge compared with unvaccinated control animals; however, the i.d. groups, including the low-dose group, appeared to have the most robust effect on disease and symptomology.

To our knowledge, this is the first demonstration of protection with an i.d.-delivered MERS, or other coronavirus, vaccine candidate. Using a sensitive RT-PCR assay, we observed significant decreases in viral loads in vaccinated animals in the lower lung regions and significant reduction in early inflammatory cytokines in response to viral infection as well as protection against symptomology. Prior work with MERS vaccine candidates has focused primarily on i.m. delivery (18, 19, 21), Additionally, this study demonstrated that a 2-dose regimen and the low-dose i.d. delivery was more impactful on disease than a higher dose i.m. delivery. In this study, we observed that i.m. delivery of synthetic DNA vaccines induced somewhat higher cellular immune responses than i.d. delivery at the same dose, though i.d. delivery induced consistent IFN-γ enzyme-linked immunospot (ELISPOT) responses. In contrast, i.d. delivery appeared to induce faster seroconversion, and higher binding antibody titers, as well as neutralizing antibody titers than i.m. delivery. This trend can be seen in this study with a MERS DNA vaccine (Figure 1) as well as in recent clinical studies of DNA vaccines targeting HIV (11) and Ebola (10). In addition, it could be that there is a different induction of T cell trafficking induced by i.d. versus i.m. immunization, such as has recently been reported in a leishmania model system (22). One hypothesis is that different cell populations are transfected between the muscle (myocytes) and skin (keratinocytes, fibroblasts, dendritic-like cells, adipocytes, and potentially some myocytes) (23), resulting in different recruitment profiles for antigen-presenting cells to the site of immunization. Additional study in this regard is warranted. i.d. delivery using synthetic DNA has significant advantages for rapid clinical development, is dose sparing with a simple administration procedure, and is associated with high tolerability.

As MERS vaccine candidates progress through preclinical and clinical studies, questions regarding animal models and efficacy endpoints are important to address. In-country human efficacy trials may be challenging due to the low number of yearly cases (<300). Data from animal models such as this NHP model may therefore have value as a bridge, with human data coming from expanded phase clinical trials. Understanding the relevance of rigorous installation challenges in NHPs will be important, as it is unlikely that humans will encounter such a high infectious dose from multiple sites. It is possible that this model is a high bar for vaccine sterilization; however, the vaccines tested in this study exhibited substantial impact and protection from disease, which was more pronounced using the i.d. route of vaccination.

The reproducibility of the NHP model of MERS-CoV infection and the clear phenotype of disease induced mimicking aspects of human infection suggests that such a model might also be useful with regards to studies of vaccines for SARS-CoV-2, the virus responsible for the COVID-19 disease pandemic that was first identified in China in 2019. Furthermore, questions have been raised by some vaccine studies in SARS and MERS challenge models reporting enhancement of viral pathogenesis in immunized animals compared with nonvaccinated controls in the absence of robust neutralizing antibodies. For example, Hashem et al. reported on an Ad5-MERS spike vaccine, which in a mouse model appeared to increase lung pathogenesis following viral challenge (24). Such enhancement of disease has also been reported for an MVA vectored spike SARS vaccine in an NHP challenge model where immunized animals presented with diffuse alveolar damage after SARS-CoV challenge, whereas control immunized animals showed only signs of minor inflammation after SARS-CoV infection (25). In the current study, no evidence of adverse lung pathology was observed in any of the dosing groups compared with unimmunized control animals. Assessment of a large panel of blood cytokines after challenge showed significant decreases in all such inflammatory mediators and were consistently observed across the animals in this challenge, suggesting that the vaccines have a benefit in prevention of virally induced destructive inflammation.

In summary, our results illustrate that a MERS spike antigen synthetic DNA vaccine administered in a 2-dose i.d. EP regimen can have positive impact in an important NHP challenge model protecting against symptoms and pathology. Dose-sparing impact was shown whereas no evidence of enhanced lung pathology and limited virally induced systemic inflammation was shown after i.d. delivery of a synthetic DNA vaccine encoding a full-length MERS-CoV spike. In addition, the vaccine induces antibody and cellular immune responses, both which can contribute to protection and clearance of virally infected cells, limiting pathogenesis and reducing viral loads in MERS-infected patients (13). Additional studies and comparison of immunogenicity data from human trials will be informative for MERS-CoV vaccines as well as for other emerging CoV infections.

## Methods

### Study design.

Groups of 6 rhesus macaques (BIOQUAL Inc.) were vaccinated twice 4 weeks apart i.m. (1 mg — 1 site) or i.d. with various doses (2 mg — 1 mg in 2 sites; 1 mg — 1 site; 0.2 mg — 0.1 mg in 2 sites) of a synthetic DNA vaccine encoding a full-length MERS-CoV S antigen with EP (6). A subset of animals (i.m., *n* = 4; i.d.-high, *n* = 4; i.d.-low, *n* = 4; control, *n* = 6) was transported from BIOQUAL Inc. to RML approximately 2 weeks before live-virus challenge. Humoral responses were similar for all selected animals, and selection was based on their cellular responses after immunization. Macaques that trended toward higher antibody and T cell levels were selected for challenge, although levels were not significantly different from animals that were not selected. Open symbols in Figure 1, C–E, indicate animals not selected for challenge. Animals were randomly assigned study numbers before arrival at RML, and all RML personnel were completely blinded to group assignments.

Rhesus macaques were inoculated with 7 × 10^6^ TCID_50_ of MERS-CoV EMC/2012 by combination of intratracheal, intranasal, oral, and ocular routes (26). After challenge, the animals were observed twice daily for clinical signs of disease and scored using a previously described clinical scoring system (the same person, blinded to group assignments, scored the animals throughout the entire study) (27). On 0, 1, 3, 5, and 6 days after inoculation, clinical exams were performed on anesthetized animals by board-certified clinical veterinarians. Blood was collected for hematology, serum chemistry, and serological analysis. Ventral-dorsal and lateral radiographs were collected. On day 6 after inoculation, all animals were euthanized, and necropsy was performed on all animals by a board-certified veterinary pathologist. Conjunctiva, nasal mucosa, mandibular lymph nodes, tonsils, pharynx, trachea, right and left bronchus, samples from all lung lobes, mediastinal lymph nodes, liver, spleen, kidney, and urinary bladder were collected for virological analysis; whole lungs were collected for histopathological analysis.

### Challenge virus.

MERS-CoV EMC/2012 (Vero passage 6) was provided by the Department of Viroscience, Erasmus Medical Center, Rotterdam, the Netherlands, and propagated once in VeroE6 cells in DMEM (MilliporeSigma) supplemented with 2% fetal calf serum (Logan), 1 mM L-glutamine (Lonza), 50 U/ml penicillin, and 50 μg/ml streptomycin (Gibco) (virus isolation medium). For inoculation of rhesus macaques, virus stock was diluted to the desired titer in DMEM.

### Hematology and clinical chemistries.

The total white blood cell count, lymphocyte, neutrophil, platelet, reticulocyte, and red blood cell count as well as hemoglobin, and hematocrit values were determined from EDTA blood with the IDEXX ProCyte DX analyzer (IDEXX Laboratories). Serum biochemistry (albumin, AST, ALT, GGT, BUN, creatinine) was analyzed using the Piccolo Xpress Chemistry Analyzer and Piccolo General Chemistry 13 Panel discs (Abaxis).

### PBMC isolation.

Whole blood was collected from each NHP into sodium citrate cell preparation tubes (CPT; BD Biosciences) containing an anticoagulant and a gel barrier. Before same-day shipment and following collection, the tubes were spun to separate and concentrate PBMCs as per the manufacturer’s instructions. Red blood cells and neutrophils pellet at the bottom of the tubes and are held in place by the gel barrier. Plasma and lymphocytes remain above the gel barrier. Each CPT can hold approximately 8 mL of blood and is shipped at room temperature. The spun CPT tubes were processed for PBMC isolation. After red blood cell lysis with ammonium-chloride-potassium buffer, viable cells were counted using ThermoFisher Countess Automated Cell Counter and resuspended in complete culture medium media (RPMI 1640 supplemented with 10% FBS, 1% penicillin/streptomycin) (R10). After removing cells for IFN-γ ELISpot and ICS assays, the remaining PBMCs were frozen in freezing media (10% DMSO, 10% RPMI, 80% FBS) in cryovials and stored long term in liquid nitrogen.

### ELISPOT assay.

To assess the cellular IFN-γ responses to vaccinations, monkey IFN-γ ELISPOT assays were performed using a IFN-γ ELISpotPRO kit (ALP) (catalog 3421M-2APW-10, Mabtech) following the manufacturer’s instructions. Briefly, 96-well plates were blocked for a minimum of 2 hours with R10 and then 200,000 PBMCs from study animals were added to each well and incubated at 37°C in 5% CO_2_ in the presence of media with DMSO (negative control), cell stimulation cocktail (PMA/ionomycin, eBioscience) (positive control), peptide pools consisting 15-mers overlapping by 9 amino acids and spanning the length of MERS S protein (GenScript, custom made), or recombinant S protein (SinoBiological). After 18–20 hours, the plates were washed and spots were developed according to the manufacturer’s instructions. Antigen-specific responses were determined by subtracting the number of spots in the DMSO containing wells from the wells containing peptides or protein stimulation.

### ELISA.

ELISA was performed to determine the antigen-specific antibody response in sera. 96-well ELISA plates (Nunc, 44-2404-21) were coated with 1 μg/ml recombinant MERS S, S1, S2, or RBD proteins (SinoBiological) in DPBS overnight at 4°C. Plates were then washed 4 times with PBS plus 0.05% Tween-20 (PBST) and blocked with 5% skim milk in PBST for 90 minutes at 37°C. After blocking buffer incubation, plates were washed and serially diluted rhesus macaque sera were added with dilution buffer (5% skim milk in PBST) and incubated for 1 hour at 37°C. Plates were washed and 1:10,000 dilution of secondary antibody HRP conjugate (4700-05, Southern Biotech, clone SB108a) was added and incubated for 1 hour at 37°C. Plates were washed, 1-step TMB (MilliporeSigma) was applied to the plates, and the reaction was stopped with 2 N sulfuric acid. Plates were then read for absorbance at 450 nm within 30 minutes using a Biotek Synergy 2 plate reader. Sera from 24 unvaccinated rhesus macaques was utilized to determine the background cut-off for calculating endpoint titers for each target protein. Sera samples were scored as positive for binding antibodies if they were 3 standard deviations above the average of the unvaccinated animals.

### Virus neutralization assay.

Two-fold serial dilutions of heat-inactivated (30 minutes, 56°C) rhesus macaque sera were prepared in DMEM containing 2% fetal calf serum, 1 mM L-glutamine, 50 U/ml penicillin, and 50 μg/ml streptomycin, after which 100 TCID_50_ of HCoV-EMC/2012 virus was added. After a 1-hour incubation at 37°C, this mix was added to VeroE6 cells. At 5 days after infection, wells were scored for cytopathic effect. The virus neutralization titer was expressed as the reciprocal value of the highest dilution of the serum that still inhibited HCoV-EMC/2012 virus replication.

### Quantitative RT-PCR.

Tissues (30 mg) were homogenized in RLT buffer and RNA was extracted using the RNeasy kit (Qiagen) according to the manufacturer’s instructions. For detection of viral RNA, 5 μl RNA was used in a 1-step real-time RT-PCR upE assay (28) using the Rotor-Gene probe kit (Qiagen) according to instructions from the manufacturer. In each run, standard dilutions with known copy numbers of a T7 in vitro–transcribed RNA standard were run in parallel to calculate the copy number of RNA present in the samples.

### Radiographs.

Ventrodorsal and lateral (right and left) radiographs were obtained using a mobile digital radiography unit with a flat-panel digital detector (Sound Technologies tru/DR) and portable x-ray generator (model PXP-HF, Poskom). Radiographs were interpreted by 2 board-certified clinical veterinarians.

### Histopathology.

Histopathology and IHC were performed on macaque lungs. Tissues were placed in cassettes and fixed in 10% neutral buffered formalin for 7 days. Tissues were subsequently processed with a Sakura VIP-5 Tissue Tek, on a 12-hour automated schedule, using a graded series of ethanol, xylene, and ParaPlast Extra. Embedded tissues were sectioned at 5 μm and dried overnight at 42°C prior to staining. Tissue sections were stained with H&E. Specific anti-CoV immunoreactivity was detected using an in-house polyclonal rabbit antibody against MERS-CoV EMC/2012 at a 1:1000 dilution. The tissues were then processed for IHC using the Discovery XT automated processor (Ventana Medical Systems) with a DAPMap kit (Ventana Medical Systems).

### Serum cytokine and chemokine analysis.

Serum samples for analysis of cytokine/chemokine levels were inactivated with γ-radiation (5 mRad) according to standard operating procedures. Concentrations of granulocyte colony–stimulating factor, granulocyte-macrophage colony-stimulating factor, IFN-γ, IL-1β, IL-1 receptor antagonist, IL-2, IL-4, IL-5, IL-6, IL-8, IL-10, IL-12/23 (p40), IL-13, IL-15, IL-17, MCP-1 and macrophage inflammatory protein 1α (MIP-1α) MIP-1β, soluble CD40 ligand (sCD40L), TGF-α, TNF-α, VEGF, and IL-18 were measured on a Bio-Plex 200 instrument (Bio-Rad) using the Non-Human Primate Cytokine MILLIPLEX map 23-plex kit (Millipore) according to the manufacturer’s instructions.

### Statistics.

GraphPad Prism 7.02/8.0 was used to analyze and plot the data. Data are presented as a range from minimum to maximum value, with all data points shown. Where appropriate, the statistical difference between immunization groups at each time point was assessed using a parametric *t* test (2 tailed) or nonparametric Mann-Whitney test, adjusted for multiple comparisons using a Bonferroni correction. Adjusted *P* < 0.05 was defined as significant.

### Study approval.

All animal experiments were approved by the Institutional Animal Care and Use Committees at BIOQUAL Inc. and at Rocky Mountain Laboratories, NIH, and were carried out by certified staff in Association for Assessment and Accreditation of Laboratory Animal Care International accredited facilities, according to each institution’s guidelines for animal use. The studies followed the guidelines and basic principles in the United States Public Health Service Policy on Humane Care and Use of Laboratory Animals (http://grants.nih.gov/grants/olaw/references/PHSPolicyLabAnimals.pdf) and the *Guide for the Care and Use of Laboratory Animals* (National Academies Press, 2011). The Institutional Biosafety Committee approved work with infectious MERS-CoV under BSL3 conditions. Sample inactivation was performed according to Institutional Biosafety Committee–approved standard operating procedures for removal of specimens from high containment.

## Author contributions

AP, ELR, HF, KM, KEB, and DBW contributed to the conception and design of the studies and reviewed data over the course of the studies. AP, ELR, FIZ, KYK, DPS, RS, FF, AO, KMW, EH, TT, RR, JL, PWH, KM, and GS performed experiments and analyzed data or generated reagents supporting the studies. JM managed the animal procurement and shipping. All authors interpreted/reviewed findings. AP, ELR, ZX, and KM contributed to the first drafts of the manuscript, HF, AP, ELR, DBW, KEB, and LMH edited sections for the manuscript. All authors contributed to and approved the final version of the manuscript.

## Supplementary Material

Supplemental data

## Version 1 04/22/2021

In-Press Preview

## Version 2 05/24/2021

Electronic publication
