# Supplementary material for: Intradermal delivery of a synthetic DNA vaccine protects macaques from Middle East respiratory syndrome coronavirus
Source: JCI Insight. 2021 May 24;6(10):e146082. doi: 10.1172/jci.insight.146082 (PMC8262283; doi:10.1172/jci.insight.146082)
Supplement: Supplemental data [file jciinsight-6-146082-s237.pdf]

Intradermal delivery of a synthetic DNA vaccine protects macaques from Middle East respiratory syndrome coronavirus

Short Title: ID Delivery of a MERS DNA vaccine in NHPs

Authors: Ami Patel[1†], Emma L. Reuschel[1†], Ziyang Xu[1,2], Faraz I. Zaidi[1], Kevin Y. Kim[1], Dana P. Scott[3], Janess Mendoza[4], Stephanie Ramos[4], Regina Stoltz[1], Friederike Feldmann[3], Atsushi Okumura[5], Kimberly Meade-White[5], Elaine Haddock[5], Tina Thomas[3], Rebecca Rosenke[3], Jamie Lovaglio[3], Patrick W. Hanley[3], Greg Saturday[3], Kar Muthumani[1,] Heinz Feldmann[5], Laurent M. Humeau[4], Kate E. Broderick[4], David B. Weiner[1]*

Affiliations:

[1]Vaccine & Immunotherapy Center, The Wistar Institute, Philadelphia, PA, USA

[2]Department of Pharmacology, Perelman School of Medicine, University of Pennsylvania, Philadelphia, PA, USA

[3]Rocky Mountain Veterinary Branch, Division of Intramural Research, National Institutes of Allergy and Infectious Diseases, National Institutes of Health, Hamilton, MT, USA

[4]Inovio Pharmaceuticals, Inc., Plymouth Meeting, PA, USA

[5]Laboratory of Virology, Division of Intramural Research, National Institutes of Allergy and Infectious Diseases, National Institutes of Health, Hamilton, MT, USA

*Corresponding Author: David B Weiner, The Wistar Institute, 3601 Spruce Street, Philadelphia, PA 19104, dweiner@wistar.org

†Authors contributed equally

Supplementary Materials:

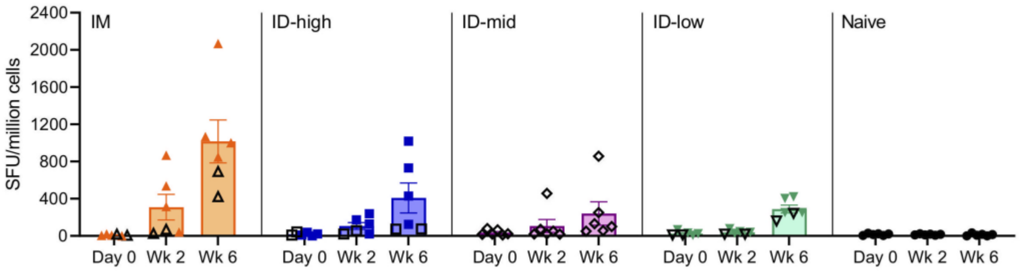

Supplemental Figure S1. Total peptide stimulated antigen-specific IFNγ ELISPOT responses (Related to Fig. 1). The total peptide stimulated IFNγ ELISPOT response for each animal is shown by the symbols with the group average indicated by the bar. Error bars are SEM. Open symbols depict the responses for animals that were not selected for challenge.

Table S1. Description of clinical signs of disease from all animals post challenge (Clinical signs of disease: Green = normal, yellow = moderate, red = severe) (Related to Figure 2A).

Group	Animal	Day 1-2	Day 3-4	Day 5-6
IM	7001	Deep abdominal breathing	Deep abdominal breathing	Deep abdominal breathing
	7045	Normal	Normal	Normal
	6839	Increased abdominal breathing, severely decreased appetite	Increased abdominal breathing, severely decreased appetite	Increased abdominal breathing, severely decreased appetite
	7032	Normal	Normal	Normal
ID-high	6827	Normal	Normal	Normal
	6848	Decreased appetite	Decreased appetite	Normal
	7037	Normal	Normal	Normal
	7024	Normal	Normal	Normal
ID-low	7005	Normal	Normal	Normal
	6880	Normal	Normal	Decreased appetite
	6824	Decreased appetite	Decreased appetite	Decreased appetite
	6997	Normal	Normal	Normal
control	7030	Decreased appetite	Increased abdominal breathing, decreased appetite	Increased abdominal breathing, decreased appetite
	6852	Irregular increased respirations, decreased appetite	Irregular increased respirations, pale, decreased appetite	Irregular increased respirations, hunched posture, pale, decreased appetite
	6831	Increased irregular respirations	Increased irregular respirations	Increased irregular respirations, pilo erection
	7014	Increased abdominal breathing, decreased appetite	Hunched posture, increased abdominal breathing, decreased appetite	Hunched posture, increased abdominal breathing, severely decreased appetite
	150129	Increased abdominal irregular respirations, decreased appetite	Increased abdominal irregular respirations, hunched posture, decreased appetite	Increased abdominal irregular respirations, hunched posture, severely decreased appetite
	6071	Normal	Deep abdominal breathing, slower	Hunched posture, quiet, irregular breathing, decreased appetite

Table S2. Summary of p values for lung viral loads between all vaccinated and control animals (corrected for multiple comparisons, p<0.05 is considered significant. Blue = significant) (Related to Fig. 2B). Parametric t-test, adjusted for multiple comparisons using a Bonferroni correction, was used for statistical analysis.

**RLL**	**LLL**	**RML**	**LML**	**RUL**	**LUL**	**R. Bronchus**	**L. Bronchus**
p=0.0061	p=0.0020	p<0.0001	p=0.0002	P=0.0225	p<0.0001	p<0.0001	p=0.0514

Table S3. Summary of p values for lung viral loads between vaccine groups and control animals (corrected for multiple comparisons, p<0.05 is considered significant. Blue = significant) (Related to Fig. 2C and 2D). Parametric t-test, adjusted for multiple comparisons using a Bonferroni correction, was used for statistical analysis.

**Group**	RLL	LLL	RML	LML	RUL	LUL	R. Bronchus	L. Bronchus
IM	p=0.0061	p=0.0249	p=0.0002	p=0.0026	p=0.0118	p<0.0001	p<0.0001	p=0.1706
ID-high	p=0.0047	p=0.0003	p=0.0025	p=0.0261	p=0.1153	p=0.0128	p=0.0311	p=0.7009
ID-low	p=0.1615	p<0.0001	p=0.0064	P<0.0001	p=0.1552	p=0.0098	p=0.0017	p=0.0243
